# Impact of Synaptic Device Variations on Classification Accuracy in a Binarized Neural Network

**DOI:** 10.1038/s41598-019-51814-5

**Published:** 2019-10-23

**Authors:** Sungho Kim, Hee-Dong Kim, Sung-Jin Choi

**Affiliations:** 1 Department of Electrical Engineering, Sejong University, Seoul, 05006 Korea; 2 School of Electrical Engineering, Kookmin University, Seoul, 02707 Korea

**Keywords:** Electrical and electronic engineering, Applied physics

## Abstract

Brain-inspired neuromorphic systems (hardware neural networks) are expected to be an energy-efficient computing architecture for solving cognitive tasks, which critically depend on the development of reliable synaptic weight storage (*i.e*., synaptic device). Although various nanoelectronic devices have successfully reproduced the learning rules of biological synapses through their internal analog conductance states, the sustainability of such devices is still in doubt due to the variability common to all nanoelectronic devices. Alternatively, a neuromorphic system based on a relatively more reliable digital-type switching device has been recently demonstrated, *i.e*., a binarized neural network (BNN). The synaptic device is a more mature digital-type switching device, and the training/recognition algorithm developed for the BNN enables the task of facial image classification with a supervised training scheme. Here, we quantitatively investigate the effects of device parameter variations on the classification accuracy; the parameters include the number of weight states (*N*_*state*_), the weight update margin (*ΔG*), and the weight update variation (*G*_*var*_). This analysis demonstrates the feasibility of the BNN and introduces a practical neuromorphic system based on mature, conventional digital device technologies.

## Introduction

Conventional computing architectures (von Neumann architectures) consume large amounts of energy when solving cognitive tasks due to the unavoidable inefficiency of data transfer between the processor and the off-chip memory. This inefficiency is referred to as the von Neumann bottleneck. Alternatively, by mimicking both the functional and structural advantages of the biological neural system, power-efficient computing systems (*i.e*., neuromorphic systems^1^) have recently been developed and are expected to offer promising breakthroughs. The practical implementation of the neuromorphic system depends on the development of ideal synaptic weight storage (*i.e*., the synaptic device). Highly integrated synaptic devices with sufficient reliability are essential for the on-chip implementation of a neuromorphic system that can process big data in real time, similar to the human brain.

Currently, various nanoelectronic synaptic devices based on two-terminal resistive switches (*i.e*., memristors) have demonstrated promising results by emulating the functionalities of biological synapses using their intrinsic analog conductance states^2–8^. Furthermore, using an integrated memristor network, functional neuromorphic systems have been experimentally applied to practical calculation tasks involving pattern recognition^9^, sparse coding^10^, matrix equations^11^, and differential equations^12^. Nevertheless, the sustainability of such devices is still in doubt due to the variability that is common to all nanoelectronic devices^13–15^. Because the physical mechanism of the conductance modulation in most prospective synaptic devices is a random process, that is, an atomic-level change based on electro/thermodynamics^16^, both cycle-to-cycle and device-to-device variations of conductance modulation are unavoidable^17^.

This concern may result from a misunderstanding of the neuromorphic system. The neuromorphic system simulates and exploits the characteristics and advantages of the brain, but this simulation and exploitation do not mean that the system must exactly imitate all of the structural and functional features of the brain. Unfortunately, with the goal of realizing a neuromorphic system that resembles the brain, most previous synaptic device studies blindly worked to demonstrate devices that were as similar as possible to biological synapses. As a result, most of the previous studies have focused only on the development/improvement of the analog conductance modulation dynamics, attempting to make them more similar to the dynamics of biological synapses while ignoring the variability issues^2–8^.

Alternatively, the sustainability and reliability of digital-type switching devices have been consistently ensured over the past 20 years^18^. Using current NAND flash technology, stable multiple memory states (4-bit = 16 states) with three-dimensional stackability have already been applied to a product. Therefore, if well-qualified conventional digital devices can contribute to a synaptic device, the aforementioned variability issues from memristors can be addressed. We have demonstrated a binarized neural network (BNN) in our previous study^19^, in which the synaptic device was a mature digital-type switchable device—a gate-all-around (GAA) silicon nanosheet transistor. By applying a supervised online training scheme, a set of multiple digital-type synaptic devices (buckets) were able to represent the analog synaptic weight. The BNN had an image classification capability that was verified by simulation and experiment^19^. However, our previous simulation was limited because the effect of synaptic device variations was ignored; the simulation was performed under the assumption that all synaptic devices in the system had equivalent characteristics without any variations. Therefore, in this study, the BNN is applied to facial image classification, and the effect of the synaptic device variations such as the number of weight states (*N*_*state*_), the weight update margin (Δ*G*), and the weight variation (*G*_*var*_) is included. The effect of device variations on the classification accuracy is analyzed quantitatively using the simulation. These results demonstrate the feasibility of BNNs, which provide higher immunity to synaptic device variability than conventional neuromorphic systems based on analog synaptic devices do.

## Results and Discussion

**Figure 1:**
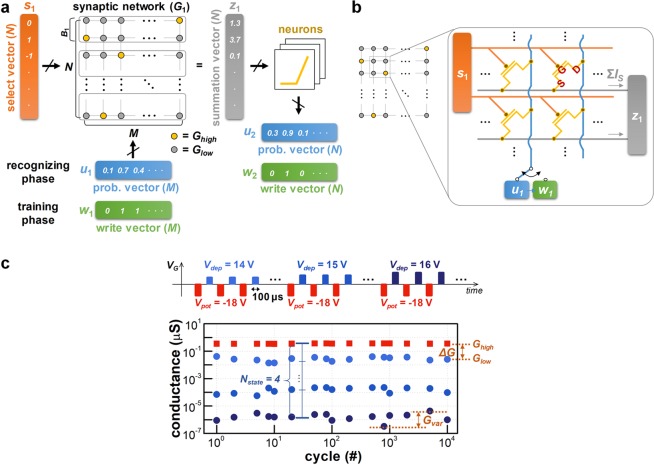
(**a**) The architecture of the binary neural network with *M* inputs and *N* outputs. The input image information corresponds to *u*_1_(*i*) and *w*_1_(*i*). The vector *s*_1_(*i*) enables supervised training by selecting a specific row, and *z*_1_(*i*) is the output of the network. (**b**) The schematic of a synaptic transistor array, where *s*_1_(*i*) involves *V*_*G*_, and either *u*_1_(*i*) or *w*_1_(*i*) involves *V*_*D*_. The integration of *I*_*S*_ along a row corresponds to *z*_1_(*i*). (**c**) Schematics of the applied pulse trains used to characterize the channel conductance modulation property of the GAA silicon nanosheet transistor. Each pulse train consists of potentiation and depression pulses applied to the gate (*V*_*pot*_ and *V*_*dep*_ for 100 μs). Control of the *V*_*pot*_ level contributes to the different conductance-switching behaviors of *N*_*state*_, Δ*G*, and *G*_*var*_.

In our previous work, we demonstrated a BNN and its supervised training scheme for an image classification application^19^. Briefly, Fig. 1a depicts the architecture of a BNN with *M* inputs and *N* outputs. The input image information is delivered into the network by two types of vectors *u*_1_(*i*) and *w*_1_(*i*), which denote the probability- and write-vector, respectively (subscripted numbers indicate the order of each network when multiple networks are involved). When an input pattern needs to be distinguished from previously trained patterns (*i.e*., recognizing phase), *u*_1_(*i*) is applied to the network. The vector *u*_1_(*i*), which is rescaled to 0 ≤ *u*_1_(*i*) ≤ 1, directly corresponds to the intensity of each pixel. When an input pattern needs to be trained by updating the synaptic weight (*i.e*., training phase), *w*_1_(*i*) is applied to the network instead of *u*_1_(*i*). The vector *w*_1_(*i*), which is defined as *w*_1_(*i*) = {0 or 1}, is stochastically determined by the learning probability *p*, defined as *p* = *γ*∙*u*_1_(*i*) (*γ* is the learning rate). Note that the most distinctive feature of the BNN is that the synaptic weights in the network *G*_1_(*i*, *j*) are given within a binary value: *G*_1_(*i*, *j*) = {*G*_*high*_ or *G*_*low*_}, where *G*_*high*_ and *G*_*low*_ represent the high- and low-conductance states of the synaptic device, respectively. To represent actual analog weights using only *G*_*high*_ and *G*_*low*_, the network *G*_1_ is partitioned into sub-buckets (the size of each bucket is *B*_1_). Each bucket is trained with a single specific input image according to the label. In addition, the selection vector *s*_1_(*i*), defined as *s*_1_(*i*) = {1 or −1 or 0}, directs the training on the input image according to the label, where 1, −1, and 0 represent “potentiation,” “depression,” and “no update” of the synaptic weight, respectively. Consequently, a set of binary values stored in the bucket can represent analog synaptic weights, which are related to the input image according to the label. Additional explanations for the operational principles of the BNN are presented in Supplementary Information Note 1.

In this study, the performance of the BNN is evaluated through the task of classifying images of faces from the Yale Face Database^20^, which contains a total of 165 grayscale images (32 × 32 pixels) of 15 individuals. In the database, there are 11 images per subject, and each image represents a different facial expression or configuration (center light, with glasses, happy; left light, without glasses, normal; right light, sad, sleepy, surprised, and winking). Here, we select 8 of the 11 images for the training set, and the remaining 3 images are used as the test set. Only the images in the training set are inputted to the network during the training phase. To evaluate the classification accuracy during the recognizing phase, only the images in the test set are inputted into the network.

For the storage of binarized synaptic weights in the BNN, a GAA silicon nanosheet transistor is used as the synaptic device (Fig. S2, Supplementary Information Note 2). The embedded charge-trap layer (silicon nitride, SiN) in the gate dielectric enables adjustable channel conductance (*i.e*., a synaptic weight update). The synaptic device array is configured such that *s*_1_(*i*) corresponds to the gate voltage (*V*_*G*_) of the synaptic transistors in a particular row, and either *u*_1_(*i*) or *w*_1_(*i*) corresponds to the drain voltage (*V*_*D*_). The source current of each synaptic transistor (*I*_*S*_) is determined by the channel conductance (*G*_*high*_ or *G*_*low*_) and *V*_*D*_. The integrated *I*_*S*_ of each row ($\sum {I}_{S}=\sum G\cdot {V}_{D}$) is the summation vector *z*_1_(*i*). Figure 1c shows the evolution of channel conductance in synaptic transistors as a pulse train is applied. Negative *V*_*G*_ (*V*_*G*_ = *V*_*pot*_) leads to the detrapping of electrons in the SiN layer, which results in an increase in channel conductance up to *G*_*high*_ (*i.e*., potentiation). In contrast, positive *V*_*G*_ (*V*_*G*_ = *V*_*dep*_) results in the decrease in channel conductance down to *G*_*low*_ (*i.e*., depression). The number of trapped electrons in the SiN layer depends on the level of *V*_*G*_. This dependence allows *G*_*high*_ or *G*_*low*_ to be adjusted, which enables control of the weight update margin (Δ*G* = *G*_*high*_/*G*_*low*_) and the multiple weight state (*N*_*state*_). The cycle-to-cycle weight variation (*G*_*var*_ = [max(*G*) - min(*G*)]/mean(*G*)) is relatively smaller even after thousands of switchings, and Δ*G* is larger than the previous two-terminal memristors whose *ΔG* is below 10 with severe fluctuations^21–24^. The remainder of the paper discusses how the improved reliability of the digital-type weight update will contribute to the sustainability of the entire neuromorphic system.

**Figure 2:**
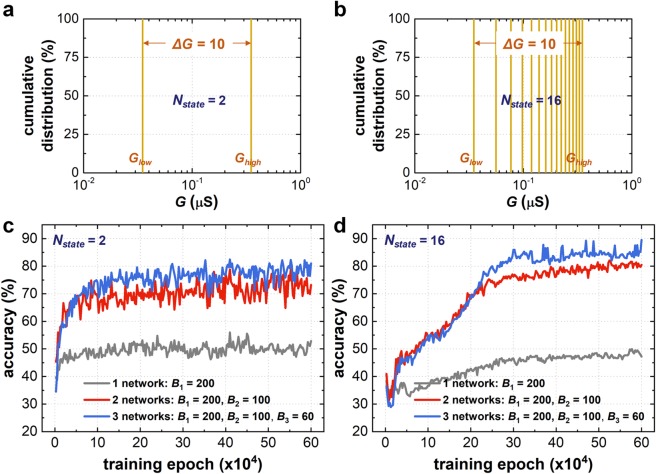
The distribution of synaptic device conductance (*G*) in the BNN when (**a**) *N*_*state*_ is 2 and (**b**) *N*_*state*_ is 16. It is assumed that there is no device-to-device variation, that is, all synaptic devices in the simulation have equivalent *G*_*high*_ and *G*_*low*_ values. The evolution of the classification accuracy as a function of the training epoch is shown for when (**c**) *N*_*state*_ is 2 and (**d**) *N*_*state*_ is 16. The simulated accuracy is obtained by repeating the simulation 10 times.

First, we investigate the impact of the number of weight states (*N*_*state*_) on the classification accuracy of the BNN. Conventional memristors can theoretically have infinite internal conductance states (*N*_*state*_ = ∞), but considering only the states that can guarantee reliability (*e.g*., data retention time or endurance), *N*_*state*_ = 8–16 is the current technological limit^25–27^. Considering this reliability limitation, *N*_*state*_ that can be obtained with current digital-type switching devices (*e.g*., *N*_*state*_ of a quad-level cell NAND flash is 16) is not inferior to memristors. To identify the effect of *N*_*state*_ on the classification accuracy in the BNN, two different cases are compared: one with *N*_*state*_ = 2 (Fig. 2a) and the other with *N*_*state*_ = 16 (Fig. 2b). The comparison assumes that there is no device-to-device variation. The simulated accuracy of facial image classification is shown in Fig. 2c,d as a function of the training epoch, where the number of networks alters the accuracy. With a single network (gray curve), the accuracy reaches approximately 50% with *B*_1_ = 200. By deploying an additional network (red curve), the accuracy improves to approximately 70% with *B*_1_ = 200 and *B*_2_ = 100. The accuracy continues to improve with more networks, up to 80% (blue curve). However, as shown in Fig. 2d, a larger *N*_*state*_ is less effective in improving the accuracy; rather, a greater number of training epochs are required. In the case of typical neuromorphic system based on the memristors, as the synaptic weight should be adjustable exactly as we desired to achieve the higher accuracy, a larger *N*_*state*_ is advantageous for more precise *G* control. However, in the case of our BNN, as binarized/quantized weight is gathered to represent a specific analog weight, the effect of the controllability in each synaptic weight on the accuracy is relatively reduced. Additionally, the pattern classification in the BNN is performed based on the bucket grouping multiple synaptic weights, and the effect of each weight value is also inevitably reduced. This unique feature of the BNN allows a feasible implementation of the neuromorphic system; engineering of the synaptic device to have a larger *N*_*state*_ is not required any more like a conventional memristor-based neuromorphic system. Consequently, although only binarized/quantized weight is used, a reasonable accuracy can be obtained from a BNN with a higher training speed. This result indicates that a neuromorphic system without analog-type synaptic weights can perform a cognitive task by exploiting both BNN architecture and its supervised training scheme.

**Figure 3:**
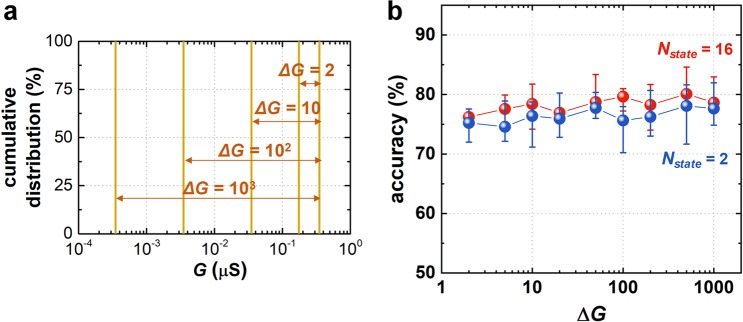
(**a**) The distribution of synaptic device conductance (*G*) in the BNN with different Δ*G*. It is assumed that there is no device-to-device variation, that is, all synaptic devices in the simulation have equivalent *G*_*high*_ and *G*_*low*_ values. (**b**) The simulated accuracy as a function of Δ*G*, which is obtained by repeating the simulation 10 times.

Next, a similar analysis was performed to study the effect of the weight update margin (Δ*G*) on the classification accuracy. In a conventional memristor-based neuromorphic system, increasing Δ*G* can improve the classification accuracy^28,29^. The Δ*G* of common memristors is about 10^21–24^, thus, much research has been devoted to further increasing Δ*G*. In contrast, our digital-type synaptic device (*i.e*., a GAA silicon nanosheet transistor) can obtain larger Δ*G* of up to 10^6^ (Fig. 1c) by modulating the amplitude of *V*_*pot*_ or *V*_*dep*_. In this BNN simulation, as shown in Fig. 3a, Δ*G* is adjusted from 2 to 10^3^, assuming no device-to-device variation. Figure 3b shows the classification accuracy as a function of Δ*G*. The modulation of Δ*G* (as well as the increased *N*_*state*_) has little effect on the accuracy, which is contrary to the behavior of conventional memristor-based neuromorphic systems. The reason for this conflicting result is as follows: The memristor-based neuromorphic system uses multiple analog states defined within *G*_*high*_ and *G*_*low*_ for image training and recognizing, and the distinguishability and stability of each analog state critically affect the performance of the system. A larger Δ*G* leads to better distinction of each analog state, resulting in better distinction between the patterns to be distinguished and the background (noise)^30^. However, since BNN uses only binarized synaptic weight values (*G*_*high*_ and *G*_*low*_), the amount of difference between *G*_*high*_ and *G*_*low*_ is not critical. Therefore, the classification accuracy in BNN is independent of Δ*G*. This feature of the BNN can be a great advantage in realizing practical on-chip neuromorphic systems, because current nanoelectronic device technology is already sufficient to produce a Δ*G* of more than 10 without any further engineering of synaptic device.

**Figure 4:**
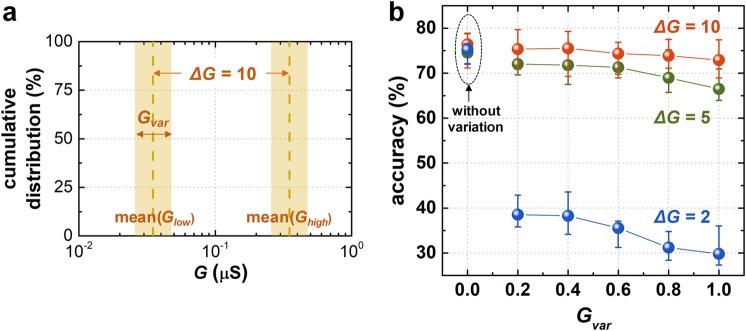
(**a**) The distribution of synaptic device conductance (*G*) in the BNN considering *G*_*var*_. The device conductance of all synaptic devices is determined stochastically within a given *G*_*var*_ range during the weight update process. Here, Δ*G* = mean(*G*_*high*_)/mean(*G*_*low*_) is fixed to 10. (**b**) The simulated accuracy as a function of *G*_*var*_, which is obtained by repeating the simulation 10 times.

Finally, the effect of the weight variation (*G*_*var*_) on the classification accuracy was analyzed. The intrinsic instability and lack of control of analog conductance switching behavior in memristors critically degrade the performance of neuromorphic systems^24,28^, although these systems are capable of tolerating device-to-device variation or noise to a certain degree. In our digital-type synaptic device, shown in Fig. 1c, *G*_*high*_ and *G*_*low*_ fluctuate during repeated switching. *G*_*var*_ can be defined as [max(*G*) - min(*G*)]/mean(*G*), where *G* is either *G*_*high*_ or *G*_*low*_. In this BNN simulation, shown in Fig. 4a, *G*_*var*_ is adjusted from 0.2 to 1.0 with fixed Δ*G* = 10. As the weight of all synaptic devices is determined stochastically within a given *G*_*var*_ range during the weight update process, this simulation considers not only cycle-to-cycle variation but also device-to-device variation. Figure 4b shows the classification accuracy as a function of *G*_*var*_. An increase of *G*_*var*_ leads to the degradation of the accuracy. When Δ*G* = 2 (blue curve), the accuracy is severely degraded, to below 40%. However, when Δ*G* is above 5 (green and red curves), the effect of an increase of *G*_*var*_ is not critical. As a BNN uses only binarized synaptic weight values, the immunity of cycle-to-cycle or device-to-device variations is considerably higher than for memristor-based neuromorphic systems. The high immunity to device variability is not the result of a well-demonstrated digital-type synaptic device. Instead, the BNN architecture and its supervised training scheme contribute to the high sustainability of the system. Therefore, further research efforts to implement a practical neuromorphic system should be devoted to developing the architecture and training scheme, rather than focusing on the improvement of analog properties in the synaptic device.

In summary, we have analyzed the impact of synaptic device variations on image classification accuracy in a BNN. The BNN has the following unique characteristics: 1) By using only binarized weight, the BNN can classify the input images with reasonable accuracy through the supervised training scheme. 2) The classification accuracy is independent of the weight update margin (Δ*G*) of the synaptic device. 3) The BNN is highly immune to variability (such as *G*_*var*_). Due to characteristics 2 and 3, current device technology is sufficient to create a synaptic device without any further research effort. Actually, prior to our study, memristor-based BNNs has been proposed to reduce the memory access by binarizing the weight^31–33^. But it is still an open question how to build and train a neural network with binarized weight. So far, each previous study has proposed different BNN operation schemes, and each study has a different point of view. The main goal of previous memristor-based BNNs is to focus on more energy-efficient processing of deep neural network algorithms. However, our research rather focuses on providing an architecture and operation scheme that is less sensitive to synaptic device variations. Consequently, our BNN can provide a device-level breakthrough for neuromorphic systems, which are currently based on conventional memristors, and provide a novel direction and inspiration for future neuromorphic engineering.

## Methods

### Test images form the yale face database

We are compliant with Yale’s policy of reuse/use of these images (http://vision.ucsd.edu/content/yale-face-database).

## Supplementary information

Supplementary Information
