# Supplementary material for: Impact of Synaptic Device Variations on Classification Accuracy in a Binarized Neural Network
Source: Sci Rep. 2019 Oct 23;9:15237. doi: 10.1038/s41598-019-51814-5 (PMC6811618; doi:10.1038/s41598-019-51814-5)
Supplement: Supplementary file 1 — Supplementary Information [file 41598_2019_51814_MOESM1_ESM.doc]

Supplementary Information for

Impact of Synaptic Device Variations on Classification Accuracy in a Binarized Neural Network

Sungho Kim^1^, Hee-Dong Kim^1^ & Sung-Jin Choi^2,*^

^1^Department of Electrical Engineering, Sejong University, Seoul 05006, Korea

^2^School of Electrical Engineering, Kookmin University, Seoul 02707, Korea

*Correspondence to S. J. C. ([sjchoiee@kookmin.ac.kr](mailto:sjchoiee@kookmin.ac.kr)).

**1. The additional explanations for the operation principle of binarized neural network**

BNN has two different modes of operation, *i.e.*, training and recognizing phases. The training phase of BNN to update the synaptic weight (Fig. S1(a)) is conducted through the cooperation of *w*_1_(*i*) and *s*_1_(*i*), which leads to three different consequences: *G*_1_(*i*,*j*) is updated to *G_high_* when *w*_1_(*i*)∙*s*_1_(*i*) = 1 (*i.e.*, *w*_1_(*i*) = 1 and *s*_1_(*i*) = 1), updated to *G_low_* when *w*_1_(*i*)∙*s*_1_(*i*) = -1 (*i.e.*, *w*_1_(*i*) = 1 and *s*_1_(*i*) = -1), and maintains its state when *w*_1_(*i*)∙*s*_1_(*i*) = 0 (*i.e.*, *w*_1_(*i*) = 0 or *s*_1_(*i*) = 0); these are referred to as ‘potentiation’, ‘depression’, and ‘no update,’ respectively. Because the higher learning probability *p* (= *γ*∙*u*_1_(*i*)) leads to *w*_1_(*i*) becoming 1 more often, the larger *u*_1_(*i*) results the potentiation/depression of synaptic weight more frequently. In terms of synaptic transistor operation, *s*_1_(*i*) = {1, -1, 0} corresponds to *V_G_* = -18 V, 15 V, and 3 V, respectively. Similarly, *w*_1_(*i*) = {0, 1} corresponds to *V_D_* = floating and 1 V, respectively. Consequently, *w*_1_(*i*)∙*s*_1_(*i*) = {1, -1, 0} leads to ‘increase’, ‘decrease’ and ‘maintain’ the channel conductance of the synaptic transistor, respectively, according to the configuration of *V_G_* and *V_D_*.

Figure S1. (a) The training phase of BNN: *G_1_*(*i*,*j*) is updated to *G_high_* when *w_1_*(*i*)∙*s_1_*(*i*) = 1, updated to *G_low_* when *w_1_*(*i*)∙*s_1_*(*i*) = -1, and maintained its state when *w_1_*(*i*)∙*s_1_*(*i*) = 0. Each element of *s_1_*(*i*) and *w_1_*(*i*) corresponds to *V_G_* and *V_D_*, respectively. (b) The recognition phase of BNN: *u_1_*(*i*) represents *V_D_*, and *z_1_*(*i*) is utilized for either classifying the input pattern or determining *u_2_*(*i*).

Next, the recognizing phase is conducted by applying *u*_1_(*i*) to the network instead of *w*_1_(*i*), as shown in Fig. S1(b) (since the weight update is not required during the recognizing phase, all *s*_1_(*i*) are set to 0). The purpose of the recognizing phase is twofold: 1) classification of the input pattern by matching with previously trained patterns, and 2) generation of *u*_2_(*i*) for transferring the input pattern information to the next network. As mentioned above, *u*_1_(*i*) involves each pixel of information of the input pattern, and the resultant *z*_1_(*i*) is the sum of *G*_1_(*i*,*j*)∙*u*_1_(*i*) in a row direction. If *z*_1_(*i*) is the output of the last network, *z*_1_(*i*) is used to classify the input pattern. The maximum *z*_1_(*i*) indicates the estimated label for a given input pattern (the detail classification process will be discussed in later). However, when multiple networks are involved in the system, *u*_2_(*i*) of the next network is generated by exploiting *z*_1_(*i*). In detail, *u*_2_(*i*) is determined by passing *z*_1_(*i*) through the designed neuron function: *u*_2_(*i*) is zero when *z*_1_(*i*) < *z*_1_(*c*), and *u*_2_(*i*) is increased linearly to 1 when *z*_1_(*i*) ≥ *z*_1_(*c*). A critical point, *z*_1_(*c*), is given according to the total number of labels (*l*). Because of the discontinuity of the neuron function, a relatively small value of *z*_1_(*i*) cannot be delivered to the next network. In other words, only meaningful information (features) of the input pattern can be transferred to the next network, which increases the classification accuracy by introducing multiple (deeper) networks. In terms of synaptic transistor operation, *u*_1_(*i*) corresponds directly to *V_D_* ranged from 0 to 1 V. Then, integrated *I_S_* row by row represents *z*_1_(*i*).

**2. The detail of gate-all-around silicon nanosheet transistor**

**2-1. Fabrication process**

Figure S2. (a) Fabrication process of nanosheet synaptic transistor. (b) Fabricated synaptic transistors on 8-inch SOI wafer. (c) Schematic of synaptic transistor and its cross-sectional view.

The fabrication process of a GAA nanosheet synaptic transistor on an 8-inch SOI wafer is shown in Fig. S2. The minimum thickness of the silicon nanosheet is approximately 5 nm, and the minimum gate length and nanosheet width are 39.6 nm and 26.4 nm, respectively. We embedded a silicon nitride (SiN) layer into the gate dielectric to enable precisely controllable channel conductance switching in a digital manner according to the charge storage in the SiN layer. Importantly, the fabrication process undergoes few divergences compared to current FinFET technologies; hence, the GAA nanosheet synaptic transistors allow the realization of a high-density artificial neural network.

**2-2. Device variability**

Figure S3. (a) Transfer characteristics of synaptic transistors measured from 242 cells. (b) Statistical performance data of synaptic transistor about I_on_/I_off_, V_T_, and subthreshold swing (SS) respectively.

Fig. S3(a) shows the transfer characteristics of the GAA nanosheet synaptic transistors measured in the entire 8-inch wafer area, which exhibits good uniformity. In addition, Fig. S3(b) shows the statistical distribution of the synaptic transistor performances. Three parameters, I_on_/I_off_, V_T_, and SS, have a good uniformity, which is superior to existing two-terminal memristors. This high uniformity with compatibility of conventional CMOS is essential for implementing a highly integrated on-chip level neuromorphic system.

**2-3. Channel conductance switching behaviour**

Figure S4. (a) Channel conductance switching behavior of synaptic transistor as a function of applied pulse width and level. (b) Switching cycle endurances of synaptic transistor.

The trapping/detrapping process of electrons in the SiN layer through the gate voltage (*V_G_*) control results in channel conductance switching. Fig. S4(a) shows the evolution of V_T_ as a function of applied pulse width and level. Red curves denote the decrease of V_T_, which indicates the increase of channel conductance (*i.e.*, potentiation). In contrast, blue curves denote the increase of V_T_, which indicates the decrease of channel conductance (*i.e.*, depression). By adjusting the width and level of applied pulse, the channel conductance can be precisely controlled. Moreover, the difference in the conductance remains uniform even with thousands of switchings as shown in Fig. S4(b), which confirms the high reliability and sustainability of digital-type weight modulation of demonstrated synaptic transistor.
